# Patient flow and cost

**Published:** 2013

**Authors:** Thulasiraj Ravilla

**Affiliations:** Executive Director: Lions Aravind Institute of Community Ophthalmology, Aravind Eye Care System, Madurai, India. **thulsi@aravind.org**

**Figure:**
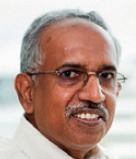
Thulasiraj Ravilla

The design of eye care services influences the ongoing operational expenses and the resources (including the costs of investing in these resources) required to deliver them. This is just as true at a hospital as it is, for example, in a district eye programme.

## District eye programmes

Here, approximately 80% of the needs for eye care services are relatively simple, such as refractive errors or cataract surgery. The remaining 20% is made up of more complex surgical interventions (including retinal or vitreous surgery, for example).

When an eye programme is well designed and well managed, solutions for simple needs (80%) can be delivered close to communities, with minimal investment and cost both to the patient and the provider.

Staff at this ‘primary’ or ‘community’ level can be trained both to meet these simple needs and to recognise the 20% of patients that must be referred. The equipment required would be the minimum needed for an eye examination, and the services provided should include refractive error correction and first-level treatment of ocular infections and injuries.

When such services are not available locally, however, the only option for patients is to go to the secondary (district or provincial) hospital or to a tertiary care facility. Then the cost goes up enormously, for both patients and providers. These centres of higher levels of care would then, for the most part, be treating conditions for which there are over-designed. They would be providing the same services with similar outcomes as in primary eye care centres, but at a significantly higher cost.

Where eye care is delivered, and by whom, has a significant impact on resources and associated costs.

## At the hospital

In a hospital setting the following factors significantly affect cost: Clinical protocol (including routine investigations)

**Figure:**
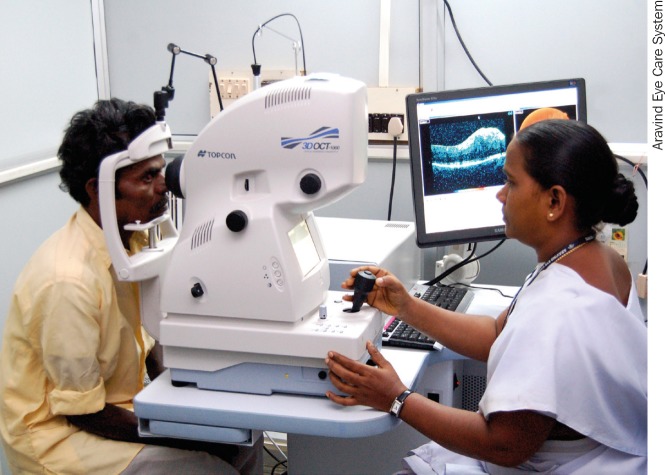
Task-shifting: fundus imaging is done by a technician.

1. Who does what in the protocol
2. Internal policy on patient flow and how different stages of care are managed.

This is best illustrated by considering two different scenarios involving a patient requiring cataract surgery.

### Scenario A

- A patient presents with severe loss of vision. On determining that cataract surgery is required, the doctor advises the patient to undergo some investigations (lab, ECG, etc.).
- The patient is advised to return on a later date to get the results and to allow the doctor to determine his or her fitness for surgery.
- The patient attends for the appointment and the doctor finds the patient fit for surgery.
- The doctor carries out the pre-operative investigations, such as keratometry and A-Scan, to determine the power of the lens to be implanted.
- The doctor or the hospital gives the patient the date for surgery, which could be several days to several months later.
- The doctor checks whether the required IOL lenses and other surgical consumables are available. If not, he or she orders them.
- The patient comes back on the appointed day for surgery and has the operation.

In this first scenario, the patient ends up making three or four visits to the hospital. Each visit triggers a series of activities such as registration, retrieving the medical record (assuming that one is maintained), repeating some of the investigations or asking the patient to provide the same information again.

The surgeon performed most of the routine clinical and administrative tasks, such as measurements for determining IOL power and ensuring its availability. This involves a lot of duplication in services as the highly paid doctor performs tasks which can be done just as well by a trained technician or manager. All of this translates into significantly higher costs.

This scenario, which also plays out regularly when getting spectacles for refractive errors, for example, illustrates that there could be enormous cost savings if we were able to accomplish much of the treatment cycle in a single visit by having appropriate policies and patient flows, as the next scenario illustrates.

### Scenario B

- A patient presents with severe loss of vision. The doctor determines that cataract surgery is required and orders the necessary investigations, as per the hospital protocol.
- All the investigations are carried out by trained technicians, including A-scan and keratometry, and the results are made available immediately.
- While the patient waits, the doctor reviews the findings. The doctor sees the patient again and advises that he or she can be admitted immediately for surgery.
- The hospital manager is responsible for ensuring that there is sufficient stock of a wide range of IOLs and that equipment is maintained regularly and kept in perfect working condition. Everything is therefore already in place for the cataract operation.
- The patient is admitted and operated on. All of this happens during a single visit.

By having a team of well-trained people who support the ophthalmologist in carrying out routine clinical and administrative tasks, costs are saved and the eye unit performs more efficiently, providing good quality care at an affordable price to more patients.

